# Facing the D‐Ilemma of Heat Resistance Parameters: From Pathogen Risk Assessment to Surrogate Selection Challenges in the Fruit Juice and Nectar Production

**DOI:** 10.1111/1541-4337.70346

**Published:** 2025-11-30

**Authors:** Astrid Gędas, Agnes Weiss

**Affiliations:** ^1^ Department of Food Microbiology, Hamburg School of Food Science University of Hamburg Hamburg Germany

**Keywords:** *D*‐value, *Escherichia coli*, *Salmonella* spp, *z*‐value

## Abstract

Over the years, numerous *D*‐ and *z*‐values have been published in the scientific literature. While these values initially appear to be valid criteria for assessing and comparing the heat resistance of different microorganisms under the same conditions or the same microorganism under different conditions, this is not always the case. This review presents a comprehensive overview of food safety in fruit processing, considering contamination routes and focusing on the selection of surrogates for key pathogens such as *Salmonella* spp. and *Escherichia coli*. Factors influencing heat resistance parameters, such as the experimental method, food matrix, mathematical model, and challenges of industrial upscaling, are explored. We critically assess the diversity and lack of methodological standardization in determining *D*‐ and *z*‐values, showing the consequences for risk assessment and surrogate selection. Along with a review of methods used by scientists, the problem of different methods for calculating the decimal reduction time is highlighted, as well as the lack of specifications for the use of *z*‐values. Additionally, this review proposes the use of the glass capillary method for testing bacterial thermal resistance in liquid foods, such as fruit juices and nectars, in order to standardize the methodology and facilitate comparisons across the literature.

## 1 Introduction

Recently, special emphasis has been placed on healthy lifestyle promotion, and thus healthier food. Next to vegetables, fruits are a major component of a balanced diet (Sivapalasingam et al. 2004). The total volume of the fresh fruit market worldwide reached 254.5 billion kilograms in 2023 and it is predicted to reach 303.70 billion kilograms by 2028 (Fresh Fruits 2024). An increasing trend can also be seen in the fruit juice industry. In Europe, the overall fruit juice volume reached 7.82 billion liters in 2022 and is forecasted to rise to 8.64 billion liters by 2027 (Statista 2023). In the United States, the total volume of juice consumed at home is expected to reach 5.18 billion liters in 2025 (Statista 2025).

To lower microbial counts and prolong shelf life, pasteurization is commonly employed in fruit juice and nectar processing. Unfortunately, applying high temperatures often leads to significant nutrient losses and undesirable changes in color and flavor (Su and Wiley 2006), both of which are critical factors influencing consumer perception (Umair et al. 2022). Studies clearly show the consumers’ preference for minimally processed products (Martins et al. 2020). Therefore, it is crucial to optimize the thermal processing of fruit juice and nectar to preserve as much of the nutritional value of the product as possible while ensuring microbiological safety.

Hazard analysis and critical control point (HACCP) principles are widely applied in the food industry to ensure safe products. The aim of using a HACCP plan is to effectively control hazards that may occur during production. For this purpose, process validation is needed, which is defined as activities other than monitoring that assess whether the system is working as planned (NACMCF 1998). A common practice is to conduct experimental trials that confirm the effectiveness of the system. Due to safety concerns and lack of adequate facilities, work with pathogens is limited to the laboratory environment, therefore nonpathogenic indicator microorganisms, known as surrogates, are used to conduct experiments under pilot plant conditions (Gurtler et al. 2010; Scott 2005). The use of surrogates in thermal treatment validations is also recommended by the Food and Drug Administration of the United States (FDA 2002). Determining the thermal resistance of a target microorganism is therefore a crucial parameter for designing effective thermal processing of the product in an industrial setting (Soni et al. 2022).

## 2 Regulatory and Microbiological Framework for Fruit Juices

According to the Codex General Standard for Fruit Juices and Nectars (CXS 247‐2005), fruit juice is defined as “the unfermented but fermentable liquid obtained from the edible part of sound, appropriately mature, and fresh fruit or of fruit maintained in sound condition by suitable means including postharvest surface treatments applied” (2005). This standard supersedes earlier individual standards, such as those for orange juice (CODEX STAN 45‐1981), concentrated apple juice (CODEX STAN 63‐1981), and apricot, peach and pear nectars (CODEX STAN 44‐1981). It specifies permissible food additives, minimum juice or puree content for fruit nectars, and recommended methods for analysis and sampling (CXS 247‐2005). Peng et al. (2017) extensively discuss food pasteurization regulations in the United States, with an emphasis on vegetables and ready‐to‐eat meals. For fruit juice production, the relevant regulation is the FDA regulation 21 CFR 120 describing HACCP Systems (CFR 2011), along with part 820 about quality system regulation (CFR 2011). In Europe, there are also regulations regarding the processing of fruit juices and nectars. The regulation (EC) No 852/2004 of the European Parliament and of the Council on the hygiene of foodstuffs presents general principles related to food chain, good hygiene practice, and compliance with procedures based also on HACCP principles. It also provides demands for food premises, with a focus on specific requirements for premises where food is located, as well as draws attention to, among others, transport, equipment, and water supply (EC 2004a). Similar to the Codex General Standard in the United States, directive 2012/12/EU of the European Parliament and of the Council specifies the definitions, such as fruit juice, fruit juice from concentrate, and fruit nectar. It defines also the composition, authorized ingredients and treatments (European Parliament and Council of the European Union 2012; 2012/12/EU).

When it comes to the standard of a 5‐log pathogen viable count reduction in fruit juice production, the FDA guideline (U.S. Food and Drug Administration 2004) only specifies that it should be “the most resistant microorganism of public health significance that is likely to occur in the juice, for example, *Escherichia coli* O157:H7” and calls it a “pertinent microorganism.” Further details can be found in regulation No 2073/2005 on microbiological criteria for foodstuffs. This regulation mandates testing unpasteurized juices for *Salmonella* spp. as absence in 25 g using the EN/ISO 6579 analytical method, and for the presence of *E. coli* according to the analytical method ISO 16649‐1 or 2. In addition, it requires the use of the ISO standard 18593 as the reference method for sampling (EC 2005). Similarly, Crandall et al. (2001) pay particular attention to *Salmonella* spp. and *E. coli* as pathogens causing outbreaks associated with fruit juices.

The shelf life of a product refers to the period during which it remains safe for consumption, meets label specifications, and maintains acceptable quality under defined storage conditions set by the food industry (Buvé et al. 2018). While traditionally determined based on experience, scientific advancements are enabling more accurate predictions of quality changes, particularly for novel processing techniques (Aaby et al. 2018; Gouma et al. 2020; Lan et al. 2021; Varela‐Santos et al. 2012; Yildiz et al. 2021). Shelf life is mainly limited by microbial growth, enzymatic activity, and chemical changes like oxidation, which are strongly influenced by processing and storage conditions (Maffe et al. 2024). For shelf‐life prediction, a useful data analysis tool could be the Integrated Pathogen Modeling Program (IPMP) developed by the USDA Agricultural Research Service (Huang 2014). According to the British Soft Drinks Association (British Soft Drinks Association n.d.), which represents UK soft drinks producers, long‐life juices typically have a shelf life of 6–12 months without refrigeration, when the pasteurization process is applied and the packaging remains sealed. In contrast, short‐life juices require chilling and last up to 30 days. In the case of unpasteurized, unprocessed juices, shelf life is considerably shorter and strongly dependent on storage temperature—approximately 16–22 days at 1°C, but only up to 8 days at 7.8°C (Fellers 1988). However, the major fruit juice market is dominated by products reconstituted from concentrate in the country of processing. The concentrate is shipped to the target market, where water is added to restore the average composition of fresh juice, and the product is then aseptically packaged into containers with a typical shelf life of up to 1 year (Ashurst 2016).

In traditional thermal treatment of fruit juices, products are heated up to 60–75°C for 30 min, followed by filling and pasteurization at 84–88°C for 15–45 min, depending on the packaging size (Renard and Maingonnat 2012). Another option is the high‐temperature short‐time pasteurization, which occurs at temperatures higher than 90°C. For instance, apple juice can be treated at 95–98°C for 15–30 s (Renard and Maingonnat 2012). To determine thermal processing conditions, decimal reduction time (*D*‐value), which is the time (min) required for a 1 log reduction of the initial bacterial population, and the *z*‐value—defined as the temperature increase (°C) needed to achieve a 90% reduction in the decimal reduction time (Singh and Heldman 1984)—are commonly used. Nevertheless, despite the long‐standing use of these parameters, inconsistencies in reported heat resistance values remain a challenge. These discrepancies arise from a lack of standardized, top‐down guidelines for research methodologies. The following sections will discuss these challenges and propose solutions for improving consistency in determining *D*‐ and *z*‐values.

## 3 Pathogens in Fruit Juice Processing

In 2022, the four most frequently reported zoonoses in humans were caused by the genera *Campylobacter*, *Salmonella*, and *Yersinia* as well as pathogenic strains of the species *E. coli* in the EU (EFSA and ECDC 2023). Similarly, in the United States, the CDC estimated in 2019 that foodborne illnesses were caused by seven major pathogens: *Campylobacter*, *Clostridium perfringens*, invasive *Listeria monocytogenes*, norovirus, nontyphoidal *Salmonella*, Shiga toxin‐producing *E. coli*, and *Toxoplasma gondi* (CDC 2025a). However, among these pathogens, in the EU the largest number of foodborne outbreaks was due to *Salmonella*, totaling in 1014 incidents. Moreover, both *Salmonella* spp. and pathogenic *E. coli* accounted for the highest proportion of hospitalizations with 38.9% and 38.5%, respectively. In comparison, campylobacteriosis contributed to 23.5% of the hospitalized cases (EFSA and ECDC 2023). It is worth noting that although enterohemorrhagic *E. coli* (EHEC) rank fourth on this list, they have the highest share of deaths and case fatalities among the pathogens mentioned (EFSA and ECDC 2023). Additionally, EHEC ranks among the most hazardous foodborne pathogens due to its remarkably low infectious dose, which may be fewer than 100 cells (Kaper et al. 2004). Besides, there is no specific therapy, and treatment primarily aims at relieving symptoms and preventing systemic complications. For that reason, prevention and control of *E. coli* infection is paramount (Lim et al. 2010).

Fresh fruits and fruit products are characterized by a high acid content, with pH range from 2.25 to 4.69 (Reddy et al. 2016), which is why they were considered microbiologically safe products for a long time (Tribst et al. 2009). However, the increasingly frequent outbreaks of foodborne pathogens, mainly EHEC and *Salmonella* spp., associated with high‐acidic products have changed this view and a new challenge for food safety has arisen (Tribst et al. 2009). It should be emphasized that *Salmonella* spp. can survive under unfavorable conditions, such as low pH values, which is why the fruit juice industry should pay special attention to it (Oyarzábal et al. 2003). This underscores the importance of adequate processing to eliminate microbial contamination. Products manufactured without the required food safety controls may pose a risk of infection with pathogens such as *Salmonella*. For instance, in April 2024, the New Zealand Food Safety Authority recalled raw fruit juices for this reason (Ministry for Primary Industries (MPI) 2025). The same applies to alternative preservation methods such as high‐pressure processing (HPP) or pulsed electric field (PEF). In October 2024, a recall of Happy Moose Juice Tropical Roots and Happy Moose Strawberry Fields was issued due to incomplete HPP treatment, which posed potential health risks from *Salmonella* spp., *L. monocytogenes*, and diarrheagenic *E. coli* (FDA 2024b).

### 3.1 *Salmonella* spp.

*Salmonella* spp. is a Gram‐negative, rod‐shaped bacterium, belonging to the family Enterobacteriaceae (Kowalska 2023). It is nonspore forming, facultative anaerobe, and able to move with flagella (Kowalska 2023; Gourama 2020). Although *Salmonella* spp strains’ optimal growth occurs at 37°C, they are able to grow in the range from 2°C to 54°C (Kowalska 2023). The genus *Salmonella* consists of two species: *S*. *enterica* and *S*. *bongori*. *S*. *enterica* is further classified into six subspecies, which are differentiated biochemically into serovars based on the composition of their capsule, flagellar, and lipopolysaccharide (LPS) structures (Hurley et al. 2014). Furthermore, in three pathogenic serotypes—namely, Paratyphi C, Dublin, and Typhi—a unique subtype of the K antigen called virulence (Vi) antigen was found (Eng et al. 2015). More than 2500 serotypes of *Salmonella* are identified yet (Eng et al. 2015; Gourama 2020). *S*. *enterica* subsp. *enterica* is the most frequently isolated subspecies from humans among all the subspecies of *Salmonella* (Eng et al. 2015). In 2020 *Salmonella* spp caused 694 foodborne outbreaks with 52.702 cases in the EU, placing it a leading foodborne pathogen (EFSA 2021). In the United States, *Salmonella* spp was responsible for the highest number of multistate outbreaks (52) and associated illnesses (2578), accounting for 66% of multistate outbreaks in 2022. The number of outbreaks showed a slight increase compared to 2021 (CDC 2025b). The infection caused by it, called salmonellosis, is characterized by such symptoms as diarrhea, vomiting, headache (Kowalska 2023). Children under 5 years old as well as adults above 60 years are at special risk of *Salmonella* infection (Eng et al. 2015). Salmonellosis is typically associated with contaminated meat products, eggs, raw milk, and undercooked poultry (Gourama 2020). However, this pathogen may also occur on fresh fruits and vegetables, especially those that have direct contact with the soil, such as strawberries (Kowalska 2023), melons, and herbs. In November 2023, a *Salmonella* outbreak occurred in the United States associated with contaminated whole and precut cantaloupe melons, resulting in 407 total illnesses, including 158 hospitalizations and six deaths (FDA 2024a). Moreover, in 2021, a multistate outbreak of *S*. Braenderup ST22 was reported, with 348 cases and 69 hospitalizations, presumed to be linked to imported melons (EFSA 2021).

*Salmonella* spp. shows high resistance to unfavorable environmental conditions, which enables their survival in the soil for a long time, even up to 216 days (Alegbeleye and Sant'Ana 2023). Microbiological diversity has a significant impact on the number of *S. enterica* in the environment. Schierstaedt et al. (2020) demonstrated that the pathogen abundance decreased in soils with highly diverse autochthonous microbial communities. In hydroponic crops, like tomatoes, strawberries or lettuce, due to direct contact between water and the edible parts of the plant, the risk of contamination of vegetables by pathogenic bacteria is even higher (Kowalska 2023). Interestingly, organic farming practices using animal manure can increase the risk of contamination by pathogens and therefore health risks (Maffei et al. 2016). Nevertheless, studies of Becker et al. (2019) and Padovani et al. (2023) demonstrated that, based on samples from South Germany and Brazil, respectively, there is no significant impact of the farming system on the *Salmonella* spp. population of produce.

*Salmonella* spp. adapt also to unfavorable environmental conditions by gaining greater resistance. Yuk and Schneider (2006) reported that 1 day adaptation in apple, orange or tomato juice leads to increased acid resistance of *Salmonella* strains, potentially enhancing survivability in a human stomach. Interestingly, acid adaptation can induce a cross‐protection mechanism against other stresses (Leyer and Johnson 1993). A relationship between bacterial culture acidification and modification in membrane fatty acid composition was noticed. For instance, Alvarez‐Ordóñez et al. (2009a) showed that higher heat resistance was observed for acid‐adapted *S*. Senftenberg CECT 4384 with low total unsaturated to saturated fatty acid ratio, and consequently low membrane fluidity. Additionally, mild heat treatment triggers the heat shock response (HSR), which enhances microorganisms' ability to withstand subsequent heat treatment (Fong and Wand 2016; Kim et al. 2021). The study of Fong and Wand (2016) showed that heat resistance of *Salmonella* spp. strains increased significantly when the cells were subjected to 45°C before a 70°C heat treatment. Therefore, it is crucial to carry out the thermal processing correctly to maintain food safety.

### 3.2 Escherichia coli

Pathogenic strains of *E. coli* may cause intestinal and extraintestinal infections, which cause around 1.7 billion cases worldwide each year (Newell and La Ragione 2018; Oloketuyi and Khan 2017; Yang et al. 2017). Pathogenic *E. coli* may be classified to several pathotypes due to their serogroups, pathogenicity mechanism, clinical symptoms, or virulence factors (Lim et al. 2010). The two major groups are the extraintestinal and the intestinal pathogenic *E. coli*. Among the extraintestinal pathogenic *E. coli* (ExPEC), the meningitis inducing *E. coli* (MNEC) and the uropathogenic *E. coli* (UPEC) are established. The intestinal pathogenic *E. coli* (InPEC) include enterotoxigenic *E. coli* (ETEC), Shiga toxin‐producing *E. coli* (STEC), enteroinvasive *E. coli* (EIEC), enteropathogenic *E. coli* (EPEC), and enteroaggregative *E. coli* (EAEC; Riley 2014), as well as diffusely adhering *E. coli* (DAEC; Kaper et al. 2004; Pakbin et al. 2021; Paletta et al. 2020). Five of these intestinal pathogenic *E. coli* including ETEC, STEC, EIEC, EPEC, and EAEC are referred to as diarrheagenic *E. coli* (DEC). Diarrheal diseases caused by DEC is one of the most significant etiological factors of diarrhea leading to dehydration and nutritional losses (Gomes et al. 2016). New technologies such as whole‐genome sequencing have recently allowed a more comprehensive view of the *E. coli* genomes and thus have blurred the strict assignation of individual strains to specific pathotypes, generating hybrid pathotypes (Newell and La Ragione 2018). One prominent example for this is the O104:H4 outbreak strain C227/11 of 2011, which was identified as a hybrid between EAEC and STEC (EFSA BIOHAZ Panel et al. 2020; Kampmeier et al. 2018; Ludwig et al. 2020).

The major pathotype of *E. coli* is STEC with 158 of the 187 known serogroups (Ludwig et al. 2020). STEC, which is synonymous to verocytotoxin‐producing *E. coli* (VTEC), from the toxin's ability to promote morphological changes in Vero tissue culture cells (Newell and La Ragione 2018), are characterized by the presence of one or more *stx* genes that encode Shiga toxins. These genes are always located on a prophage (Zhang et al. 2020). Virulence of *E. coli* is very complex, which is why it consists of many factors. Pakbin et al. (2021) extensively described the virulence factors of *E. coli* pathotypes and differentiated four principal virulence groups, which are colonization, fitness, toxins, and effectors. Strains that were isolated from patients and found to cause hemorrhagic colitis and the hemolytic‐uremic syndrome are called enterohemorrhagic *E. coli* (EHEC; Yang et al. 2017). The EHEC pathotype is the main pathogenic agent found in contaminated food matrices, such as fruit and vegetables (Enciso‐Martínez et al. 2022). In report of the EFSA and the European Centre of Disease Prevention and Control from 2019, the most common serogroups were O26 (38.5%), O157 (23.0%), O80 (9.0%), and O145 (8.0%) among all hospitalized cases (EFSA 2021). The biggest outbreak of STEC O111 associated with apple cider took place in New York (Schaffzin et al. 2012). On September 28th, 2004, a local community hospital notified four patients hospitalized with diarrheal illness. Results of the epidemiological investigation identified a final number of 213 cases in total and identified the source of the outbreak as the consumption of unpasteurized apple cider from a local orchard that was a popular public attraction (Schaffzin et al. 2012).

Pathogenic *E. coli* are present in diverse environments. They inhabit water, soil, plants as well as animal gastrointestinal tracts, especially those of ruminants such as cattle, sheep, and goats. For this reason, mankind is often exposed to this pathogen (Oloketuyi and Khan 2017). Infection with *E. coli* can be due to direct contact to infected animals, contaminated water or person‐to‐person transmission (Mulchandani et al. 2021). However, the most common route of infection is ingestion of contaminated food (52%; Lim et al. 2010; WHO 2018). In 1982, after two food‐related outbreaks in the United States with 26 cases in Oregon and 21 cases in Michigan, *E. coli* O157:H7 was for the first time described as a pathogen that causes bloody diarrhea and renal failure (Doyle 1991). In 1985, 27 cases of diarrhea were reported in England after a social function where a cold buffet was served (Riordan et al. 1985). In 2011, the first outbreak of *E. coli* O157:H7 with fresh strawberries from roadside stands or farmers’ markets as vehicles was reported in the United States. Fifteen cases were identified, from which six were hospitalized, including four with hemolytic uremic syndrome (HUS), and two people died. The investigation confirmed that the berries were from a single farm, Jaquith Strawberry Farm, located in Newberg, and were contaminated with deer feces (Laidler et al. 2013; OHA 2011). This highlights that fruits growing close to the ground and exposed to wild animal feces are at increased risk of *E. coli* contamination. This was not the only time that feces of wild animals was considered as the potential contamination source. For instance, in the 1996 outbreak caused by unpasteurized apple juice dropped, contaminated apples were used in the juice production (CDC 1996; Cody et al. 1999).

Additional to outbreaks in which people became ill or even died after consuming contaminated products, many times there are also situations where products are withdrawn from the market because foodborne pathogens were detected. For instance, in 2014 in Ontario, Canada, companies such as Rolling Acres Cider Mill, Osoleo Wildcrafters, and Noah Martin were recalling their products unpasteurized apple, cranberry‐apple, and pear cider due to *E. coli* O157:H7 contamination (CFIA 2014a, 2014b, 2014c).

### 3.3 Contamination Routes

**FIGURE 1:**
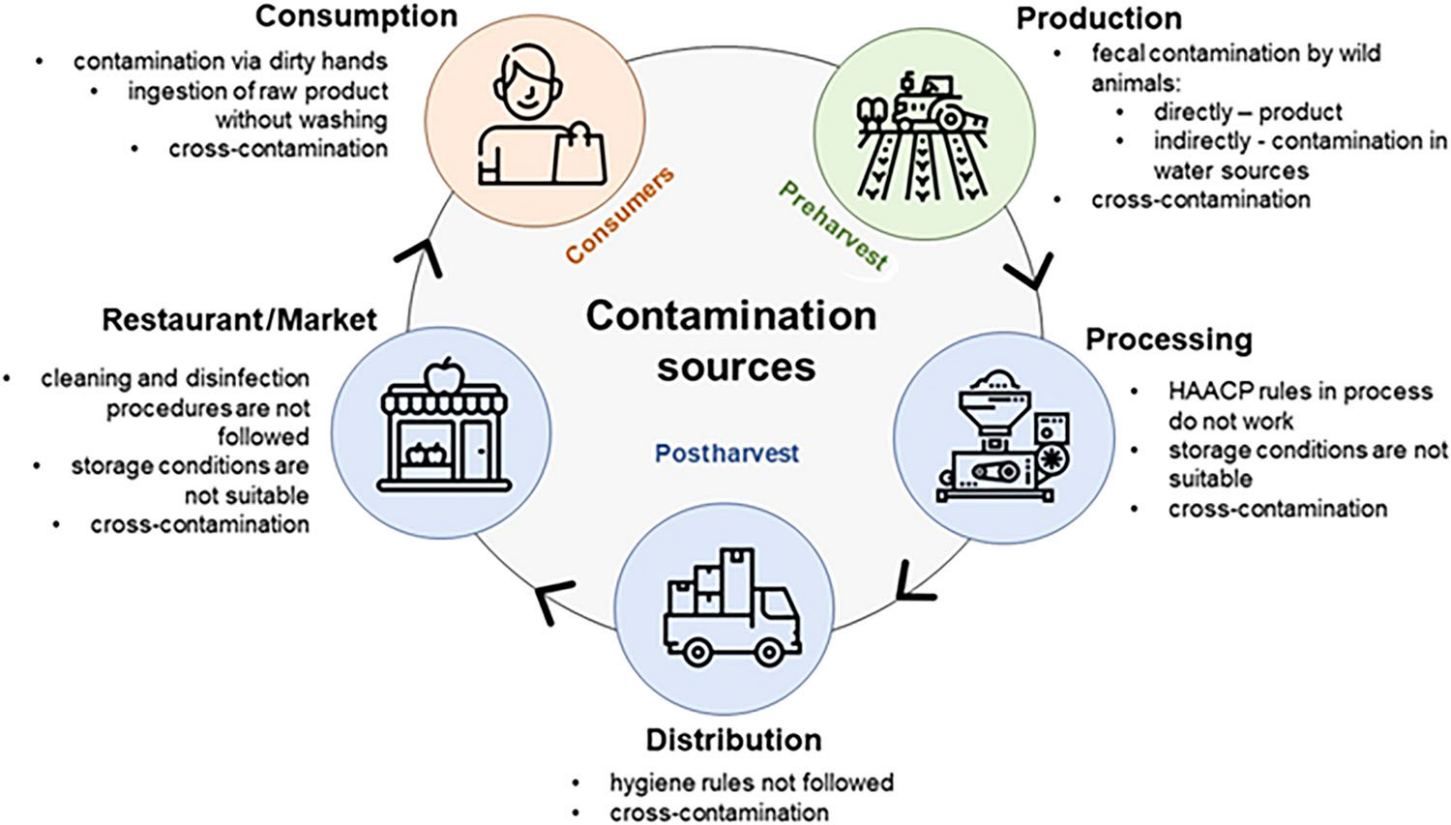
Contamination sources and mechanisms in the fruit production chain (based on: Jay‐Russel 2013; CDC 2024).

Identification of possible transmission routes of foodborne pathogens in the food supply chain is the key to avoid or minimalize contamination of the product. The main stages in the food supply chain are common for most products and these are: production, processing, distribution and preparation (CDC 2024). Figure 1 shows the supply chain using the fruit juice and nectar production as an example with identified risk factors at each stage, divided into pre‐ and postharvest stages. During the first stage, the preharvest phase, pathogens can colonize growing crops (Berger et al. 2010). Major contamination sources are the plant environment and other fruits (Al‐Zenki et al. 2012). However, as Bennett et al. (2015) pointed out using tomatoes as an example, there is often a lack of appropriate labeling, and the practice of mixing products from different origins during repackaging and distribution complicates traceback and subsequent source investigations. Additionally, water that may run off from nearby pastures may be microbiologically contaminated (Berger et al. 2010). Due to the outbreaks caused by fecal contamination of plants, it is considered as the significant risk factor at the preharvest production stage (Jay‐Russell 2013). Wild animal feces may spread pathogens directly onto the plant or may contaminate it indirectly via water and soil. Thus, the risk of contamination is especially high for crops grown close to the ground, for example, strawberries.

To ensure microbiological safety in a fruit processing plant, all critical processing factors must be controlled to minimize the risk of contamination (Al‐Zenki et al. 2012). Although most juice sold in the United States is heat‐treated, certain grocery stores, health food stores, cider mills, farmers’ markets, and juice bars sell packaged juice made on‐site that has not been pasteurized or otherwise treated to ensure its safety (FDA 2015). It should be emphasized, however, that the lack of heat treatment or alternative processing carries a greater probability of contamination. Therefore, FDA (2015) requires warning labels to draw consumers' attention to this potential risk. With regard to EU regulations, Regulation (EU) No 853/2004 includes only specific requirement of additional labeling of “raw milk” (2004b). Further, cross‐contamination can occur at any stage, for example, through other carriers such as agricultural equipment or workers clothing. Hence, a strict control of the whole process of fruit processing, including transportation, distribution, storage, and handling is very important. Kothe et al. (2019) assessed the growth of pathogenic bacteria on fruits and vegetables, which are often served at buffets in restaurants, at different temperatures. They reported that five strains of *E. coli* (CQ isolated from a hot dog, ECHC isolated from human cystitis, DH5α, ATCC 8739, and ATCC 25922) cannot grow at 10°C, however they are able to grow fast on papaya and watermelon at 20°C in 4 h, and at 30°C even in 2 h. This result underlines the importance of the storage conditions of fruits. Interestingly, there was an absence of *S. aureus* growth on tomatoes and papaya, which may be explained by the low pH values of tomatoes and the high content of proteolytic enzymes in papaya. In contrast to that, *E. coli* was able to grow on this food, even if it is considered as an unfavorable environment for microbial growth, which underscores the necessity to develop and monitor pasteurization procedures concerning these organisms. Strawn and Danyluk (2010) showed that *E. coli* O157:H7 can survive on cut pineapple for the shelf life at each of the tested temperatures, namely, 23, 12, 4 and −20°C. Other investigations indicate that although *E. coli* O157:H7 as well as *Salmonella* spp. do not grow on frozen strawberries, they may survive on the fruit for periods longer than 1 month (Knudsen et al. 2001). These studies confirm that not only fresh, but also processed fruit can be a potential vector of pathogen transmission and pose a risk of contamination.

## 4 Roles of and Selection Criteria for Surrogate Microorganisms

Nonpathogenic microorganisms that exhibit a similar behavior as pathogens under the same treatment conditions and mimic their survival responses can be defined as surrogates (FDA 2013). These microorganisms may be used as biological indicators, due to their similar properties, such as growth kinetics or resistance to stress conditions. Surrogates are generally used to replace pathogens that would pose a potential risk to public health if directly used, like *Bacillus anthracis* classified as a biosafety level 3 microorganism (Greenberg et al. 2010), or pathogenic *E. coli* and *Salmonella* spp. (Woerner et al. 2018). Unlike their pathogenic counterparts, surrogates can be safely introduced into the food production areas to assess the efficacy of viable count reduction, cleaning, or disinfection steps (FDA 2001; Busta et al. 2003; Kim and Harrison 2009). This practice is particularly beneficial for companies and academic institutions that do not have access to biosafety level 2 (BSL‐2) laboratories (Hu and Gurtler 2017).

**FIGURE 2:**
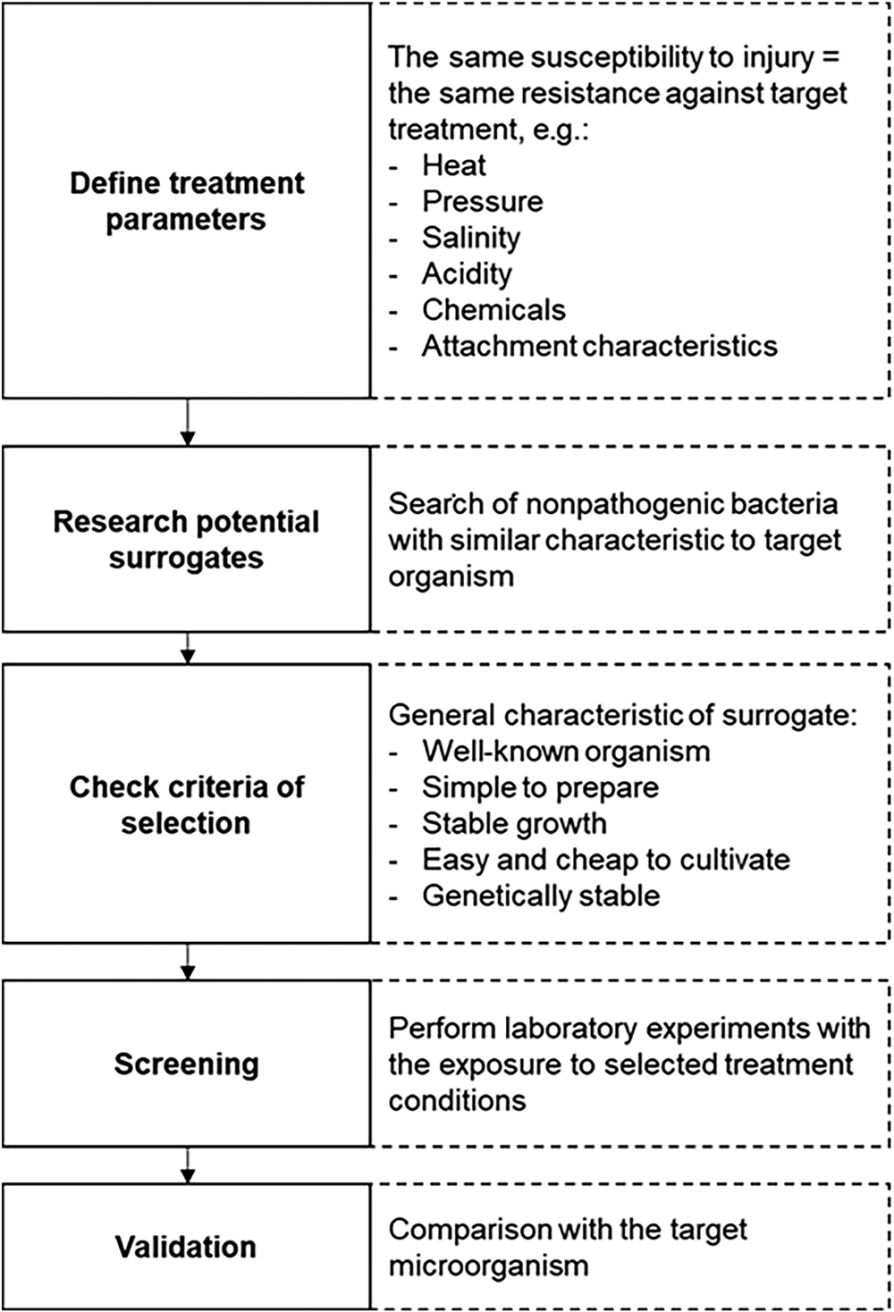
Surrogate selection workflow (based on: Hu and Gurtler 2017).

The selection process of a suitable surrogate for fruit juice and nectar processing is carried out in a step‐wise procedure according to the scheme shown in Figure 2. This process is crucial for the validity of the results, as microorganisms react differently to diverse stresses. The parameters of the applied treatment and the target pathogen must first be defined. The possible treatments are not limited to heat treatments, but include for example pressure treatment or chemical disinfection. Therefore, the surrogate candidate must have the closest possible resistance to the selected type of stress as the target pathogen. For this purpose, experiments are conducted to evaluate the resistance of selected microorganisms to a specific stress factor, such as heat, pressure, sanitation, or chemicals, and the results are then compared to the tolerance of the target pathogen. It needs to be stressed that different surrogate strains may be used for one pathogen for various treatments. It should be noted that pathogenic strains are frequently characterized by unique properties which enhance their resistance and virulence. For instance, *E. coli* O157:H7 has a strong acid tolerance, and thus can survive in environments with low pH values like the stomach (Eblen et al. 2005). Extensive studies are needed to identify suitable surrogate microorganisms that meet different spectra of requirements. The screening results of selected studies in fruits and fruit products are shown in Table 1. As can be seen there, a wide range of different strains was suggested for diverse fruit matrices and treatment conditions even for the same pathogen. For example, for *E. coli* O157:H7 the most frequently indicated surrogate bacterium is *E. coli* ATCC 35218 for PEF or radio frequency electric field, while for UV light and pulsed light treatment, the indicated strain is ATCC 25922.

**TABLE 1:** Determined surrogates for *Salmonella* spp. and *E. coli* O157:H7 used for validation tests of different inactivation methods in fruit, fruit juices, and nectars.

Target pathogen	Number of selections	Selected surrogate	Food matrix	Applied inactivation method	References
*Salmonella* spp.	2	*Enterococcus faecium*	Citrus surface (lemon, orange)	Finishing wax	Wang et al. 2024
			Apple pieces	Hot‐air drying	Grasso‐Kelley et al. 2021
	2	*E. coli* ATCC 11229	Strawberry nectar	Thermal treatment	Gędas et al. 2024b
			Orange surfaces	Alkaline washing	Pao and Davis 2001
	2	*E. coli* cocktail (ATCC 25922 and ATCC 35218)	Grapefruit surface	Survival during 14‐day storage	Danyluk et al. 2019
				Brush wash system	Danyluk et al. 2019
	1	*E. coli* ATCC 25922	Apple surface	Hydrogen peroxide decontamination	Eblen et al. 2005
	1	*E. coli* ATCC 25253	Apple surface	Hydrogen peroxide decontamination	Eblen et al. 2005
*E. coli* O157:H7	5	*E. coli* ATCC 35218	Orange juice	Pulsed electric field (PEF)	Gurtler et al. 2010
			Strawberry juice	Pulsed electric field (PEF)	Gurtler et al. 2011
			Cranberry juice	Radio frequency electric field (RFEF)	Rezaeimotlagh et al. 2018
			Strawberry purée	Pulsed electric field (PEF)	Geveke et al. 2015
			Orange juice	Pulsed electric field (PEF)	Sampedro et al. 2013
	5	*E. coli* ATCC 25922	Pomegranate juice	UV light	Pala and Toklucu 2021
			Apple juice	Pulsed light treatment	Sauer and Moraru 2009
			Apple cider	Pulsed light treatment	Sauer and Moraru 2009
			Apple cider	UV light	Duffy et al. 2000
			Apple juice	UV light	Usaga et al. 2016
	3	*E. coli* ATCC 8739	Apple juice	UV‐C light	Orlowska et al. 2015
			Apple juice	Pulsed electric field (PEF)	Evrendilek et al. 1999
			Strawberry nectar	Thermal treatment	Gędas et al. 2024a
	3	*E. coli* K12	Tropical fruit smoothie (pineapple, banana, coconut milk)	Combination of heat and pulsed electric field (PEF)	Walkling‐Ribeiro et al. 2008
			Apple juice	Shear stress, moderate electric field and nisin	Mok et al. 2020
			Apple juice Orange juice	High intensity light pulses	Palgan et al. 2011
	2	*E. coli* ATCC 11229	Orange surfaces	Thermal treatment	Pao and Davis 2001
			Orange surfaces	Alkaline washing	Pao and Davis 2001
	1	*E. coli* ECRC 97.0152	Apple surface	Hydrogen peroxide decontamination	Eblen et al. 2005

In each case, the decisive factor is tolerance to the stress condition in question. The aim is to identify a nonpathogenic microorganism with comparable or closely matching tolerance. Gurtler et al. (2010) investigated a suitable surrogate for *E. coli* O157:H7 during PEF treatment of orange juice by comparing microbial inactivation under identical conditions. Bacteria were exposed to 20 kV/cm at 55°C for 70 µs. *E. coli* ATCC 35218 was identified as a suitable surrogate, as its inactivation rate—2.02 log CFU/mL—was comparable to that of the tested EHEC strain—2.22 log CFU/mL (Gurtler et al. 2010). Once the requirements for the applied treatment are established and surrogate candidates are identified through a literature review, they should be checked in the next step to ensure they meet the following general criteria. The surrogate must fulfill several criteria—it should be a well‐known organism, simple to prepare, exhibit stable growth, be easy and inexpensive to cultivate, and remain genetically stable (Hu and Gurtler 2017; FDA 2001; Busta et al. 2003). A phylogenetic relative is likely to be more morphologically and behaviorally similar to the target organism (Greenberg et al. 2010). Therefore, attention should be paid to the genetic relationship. However, this is not a necessary condition. Wang et al. (2024) showed *Enterococcus faecium* as a suitable surrogate for *Salmonella* spp. in finishing wax on citrus surfaces. In this case, both microorganisms come from different families—Enterobacteriaceae and Enterococcaceae—and yet they show great similarity in terms of resistance to the applied treatment. A further crucial point is the safety assessment regarding the risks associated with surrogate microorganisms. Therefore, surrogate candidates should be tested for virulence factors (Hu and Gurtler 2017). For this purpose, tools such as VirulenceFinder 2.0 (Malberg Tetzschner et al. 2020; Joensen et al. 2014) or Bacterial and Viral Bioinformatics Resource Center (BV‐BRC, https://www.bv‐brc.org/) might be helpful to ascertain whether the tested bacterium may be hazardous or if the potential virulence factors are only hypothetical proteins. This especially applies if the selected potential surrogate strain belongs to a genus which is known to incorporate pathogenic strains, such as *Enterococcus* spp. In the next step, surrogate candidates should be evaluated including performing lab‐scale experiments to establish their resistance against the selected inactivation factor. For instance, in conventional fruit juice and nectar processing, bacterial thermal resistance is most relevant, since pasteurization is a common treatment to prolong the product's shelf life. Microorganisms with lower resistance as the target pathogen may give false predictions and in consequence lead, for example, to inadequate thermal treatment. Therefore, for in‐plant indicators bacteria with greater survival properties are chosen to ensure a margin of safety (Hu and Gurtler 2017). However, this can lead to unnecessarily long processing, during which the valuable nutritional value of the fruit product is lost and it adversely affects the organoleptic quality of the product as well. For these reasons, it is extremely hard to find nonpathogenic bacteria which will correlate with the target processing environment. The selection of a surrogate should be finally evaluated by comparing the behavior with the pathogen under the given conditions (Hu and Gurtler 2017).

## 5 Challenges in Microbial Thermal Resistance Determination

In thermal processes, the industry commonly relies on thermal resistance data obtained from scientific literature as a crucial component of the validation process (Scott 2005; Ceylan et al. 2021), as well as on practical experience. The basic parameters of the thermal resistance of microorganisms are the *D*‐ and *z*‐value. The *D*‐value is calculated according to Equation (1) (Singh and Heldman 1984).

(1) $$D=\frac{t}{\log N_{0}-\log N}$$

where *t* (min) is the time, log*N*_0_ (cfu/mL) is the logarithmic initial population, and log*N* (cfu/mL) is the logarithmic final population.

The *z*‐value can be expressed by Equation (2) (Singh and Heldman 1984).

(2) $$z=\frac{T_{2\;}-T_{1}}{\log D_{T_{1}}-\log D_{T_{2}}}$$

where *T*_1_ and *T*_2_ (°C) are the temperatures, and log*D_T_*_1_ and log*D_T_*_2_ are the corresponding logarithmic *D*‐values.

In the food processing industry, the lethality value (*F*‐value) is often used (IFU 2024). The result of multiplying the *D*‐value of the selected target microorganism at a selected temperature by the amount of required decimal reduction is called the *F*‐value (Renard and Maingonnat 2012). For example, there is a need to follow a “5D” concept, which applies to *E. coli* O157:H7, with a *D*‐value at 57°C in cantaloupe juice of 8.2 min (Sharma et al. 2005). The corresponding *F*‐value is calculated as follows: 5 × 8.2 min = 41 min. Calculations of the lethal effect of heat on microorganisms at a selected temperature are based on the *D*‐ and *z*‐values. Therefore, the following sections of this review will focus on these parameters.

### 5.1 Variety of Methods for *D*‐Value Measurement

The *D*‐ and *z*‐values are the main parameters used to set the process and thermal treatment of the product in the industry and its optimization. This is the basic conventional model that only takes the temperature into account. However, the thermal resistance of bacteria depends on many different factors. Soni et al. (2022) also drew attention to problems related to variation in *D*‐values reported in the literature. One of them is the chosen method in estimating the thermal resistance of a given microorganism. To highlight the variety and heterogeneity of currently published methods, Table 2 gives an overview of the different methods that have been used by researchers for determining the *D*‐value so far.

**TABLE 2:** Methods of microbial thermal resistance determination.

Nos.	Method	References
1	Capillary tube	
	20 µL	Usaga et al. 2014a 2014b
	40 µL	Splittstoesser et al. 1996
	50 µL	Sörqvist 1989; Fujikawa et al. 2000; Sharma et al. 2005; Topalcengiz and Danyluk 2017; Gędas et al. 2024a
	100 µL	Casadei et al. 2001
2	Glass tube	
	1.5 mL	Gędas et al. 2024a, 2024b
	2 mL	Sörqvist 1989; Fujikawa et al. 2000
	9.9 mL	Pagal and Gabriel 2020
3	Screw‐capped test tubes—2 mL	Gabriel 2007
4	Thermal‐death‐time disks (TDT disks)—1 mL	Yuk et al. 2009; Jin et al. 2008; Lau et al. 2021
5	Thermal‐death‐time tubes—1 mL	Büchner et al. 2012
6	TDT Sandwich	Lau and Subbiah 2020; Lau et al. 2021
7	TDT cells	Chung et al. 2008; Tsai et al. 2019
8	TDT II cells	Jin and Tang 2019; Sun et al. 2023; Xie et al. 2021
9	Three‐neck flask—150 mL	Mazzotta 2001
10	250 mL Erlenmeyer flask—100 mL	Ryu and Beuchat 1998; Enache et al. 2006
11	Thermoresistometer Mastia—350–400 mL	Alvarez‐Ordonez et al. 2009b; Conesa et al. 2009; Huertas et al. 2021; Clemente‐Carazo et al. 2020
12	Submerged coil heating apparatus—10 mL	Cole and Jones 1990; Enache et al. 2011
13	Vacuum‐packed sterile pouch	
	8 × 8 cm	Evelyn and Silva 2015; Lau et al. 2021
	10 × 5 cm	Suhalim et al. 2023
	Stomacher 80 bag (3 g)	Kenney and Beuchat 2004
	Stomacher 400 bag (100 g)	Juneja et al. 2001
	7 × 13 cm (10 g, ∼1–2 mm thick)	Byrne et al. 2006
	7.5 × 12.5 cm	Kim et al. 2019
14	Stainless steel containers—300 g	Szpinak et al. 2022

The most frequently used method uses glass capillaries heated in a water bath (Casadei et al. 2001; Fujikawa et al. 2000; Sharma et al. 2005; Sörqvist 1989; Splittstoesser et al. 1996; Usaga et al. 2014a; 2014b; Topalcengiz and Danyluk 2017). According to this method, the tested sample with a volume of 20–100 µL is placed in a glass capillary, which is sealed with fire, and then immersed in a water bath at the selected temperature. Several to a dozen of such capillaries are used in one experiment, depending on how accurate and how long the measurement is to be. The tested capillaries are removed one by one at established time intervals and placed in an ice bath for subsequent viable count analysis. This method has the indisputable advantage that heat transfer through the thin capillary wall occurs faster than in standard glass tubes. Although this method is considered the best, due to problems with placing the sample in a narrow gap it is only suitable for testing fluids (Chung et al. 2008) such as apple juice (Splittstoesser et al. 1996; Usaga et al. 2014a) or orange juice (Topalcengiz and Danyluk 2017).

A solution for heating solid food in a water bath may be the use of plastic bags. Juneja et al. (2001) tested this procedure in the experiment of thermal inactivation of *Salmonella* spp. in poultry and used Stomacher 400 plastic bags. The inoculated chicken meat was blended and the samples in the bag were compressed into a thin layer and sealed, and so prepared, they were ready for thermal inactivation in the water bath. The heat‐sealed bags can also be used when testing creamy and pasty products such as peanut butter (Kenney and Beuchat 2004) or various types of cookie dough (Suhalim et al. 2023).

Unfortunately, in some cases these methods may not meet restrictive safety regulations. These safety regulations vary depending on the country and the specific bacterial strains being studied. For example, in Germany, experiments involving EHEC strains require a biosafety level 3 (TRBA 2015) laboratory, which imposes special safety rules to be followed. For instance, one guideline stipulates that devices coming into contact with biological materials must be capable of disinfection or washing (Biological Agents Ordinance 2013). Those that cannot meet this criterion should not be used in the level 3 area. Additionally, it is crucial to assess how the chosen experimental method impacts on environmental contamination. When handling organisms classified under risk group 3, which pose severe human health risks, precautions must be taken to minimize contamination. Thus, plastic bags may be risky due to the potential for tearing or leaking at the seal, which might occur unobserved and thus pose a major risk for the lab personnel.

Taking this into consideration, several researchers have chosen the experimental method using glass tubes (Gędas et al. 2024a, 2024b; Fujikawa et al. 2000; Pagal and Gabriel 2020; Sörqvist 1989). This method is suitable for testing liquid foods such as strawberry nectar (Gędas et al. 2024a, 2024b) or orange juice (Pagal and Gabriel 2020), which may also contain fruit pulp. The procedure is the same as in the case of a glass capillary, several samples are placed in glass tubes and then heated in a water bath. The clear difference is the volume of the sample, which can be 1.5 mL (Gędas et al. 2024a, 2024b), 2 mL (Fujikawa et al. 2000; Sörqvist 1989) or 9.9 mL (Pagal and Gabriel 2020). However, the results obtained with this method differ from those using the glass capillary method. For example, thermal resistance of *E. coli* K12 was up to 4.5 times higher when measured using glass tubes compared with glass capillaries (Chung et al. 2007). Gędas et al. (2024b) similarly observed that *E. coli* ATCC 8739 had up to 6.6 times higher *D*‐values when using glass tubes. The disadvantage of using glass tubes, which also explains the higher *D*‐values, is a longer come‐up time, which increases with the sample volume. At the industrial scale, thermal treatments are typically performed using tubular or plate heat exchangers. These systems provide a high surface area‐to‐volume ratio, enabling rapid and uniform heating of the product and thereby reducing both come‐up and residence times (Huang et al. 2020). This not only improves process consistency and energy efficiency, but also reduces the risk of under‐ or overprocessing, thereby supporting both microbial inactivation and the preservation of product quality. A solution in this case in laboratory conditions may be preheating of the samples and then adding the bacterial inoculum in as small a volume as possible only after reaching the desired temperature. This approach was used, for instance, by Gędas et al. (2024a, 2024b) and Pagal and Gabriel (2020). The advantage of this method is certainly the possibility of testing liquids with higher density or viscosity, as well as liquids with solid particles, like cloudy juice, which could block the narrow entrance of the glass capillary.

Another frequently used method is the thermal‐death‐time (TDT) cell. The test cell, designed by Chung et al. (2008), was constructed of aluminum, which is characterized by high thermal conductivity, great resistance against corrosion, and machinability. These researchers also wanted to obtain a test cell that would provide a hermetic seal to allow a sample heating above the boiling point of water (Chung et al. 2008). The disadvantage of this method was a long come‐up time. For this reason, Jin and Tang (2019) improved the design of TDT cells. These researchers were able to significantly reduce the come‐up time from 2.33 min to 0.67 min with a brown rice flour sample. However, this method is adapted to low water activity foods, like ground cinnamon (Xie et al. 2021) or cocoa powder (Tsai et al. 2019), where a closed testing environment is very important to reduce the risk of changes in moisture content.

Another factor influencing the *D*‐value may be the way of heating itself, such as a heating block in comparison to a water bath. De Souza Figueiredo et al. (2019) highlighted that the chosen method can significantly influence the results. In that study, *D*‐values for STEC strains, obtained when heating 2 mL micro tubes in a water bath and in a heating block at 60°C, were compared. The heating block had a higher come‐up time as well as the standard deviations between duplicates were approximately 10 times higher than with the water bath method (De Souza Figueiredo et al. 2019).

To optimize the determination of the bacterial thermal resistance, some researchers design the containers to hold the sample themselves. Büchner et al. (2012) designed a fast‐responding aluminum tube that has a 15 s come‐up time, which allows for a very accurate measurement of the thermal inactivation only at the target temperature. It is similar to the measurement device thermoresistometer Mastia, which was built by the team of Conesa et al. (2009). Some scientists emphasize the advantages of dry heating, such as in the TDT Sandwich, compared to wet heating, such as used in a water bath (Lau and Subbiah 2020). Unfortunately, while these methods may be considered better experimental setups, they are not commercially available, making it difficult for other research teams to use them in their studies. The most common device for thermal treatment testing is still a water bath. However, when testing endospores of bacteria such as *Clostridium perfringens* at temperatures above 100°C, an oil bath is used (Bradshaw et al. 1977).

Developing new research methods or enhancing existing ones is a common practice in the scientific community. However, in the case of research on the thermal resistance of microorganisms, this has not resulted in uniform or comparable results. Based on the reviewed studies, glass capillaries provide the fastest heat transfer and are particularly suitable for homogeneous liquids. They are frequently used for testing fruit juices (Gędas et al. 2024a; Sharma et al. 2005; Splittstoesser et al. 1996; Topalcengiz and Danyluk 2017; Usaga et al. 2014a) and represent a relatively simple, widely accessible method that avoids the risk of scientific exclusion. Therefore, this method is recommended for research on the thermal resistance of microorganisms in fruit juices and other beverages. Nonetheless, glass capillaries pose challenges for pulpy or viscous food products, such as smoothies or fruit purees. For such matrices, glass tubes should be considered. However, further research is required to address discrepancies in the come‐up time associated with this method.

### 5.2 Influence of the Food Matrix on *D*‐Values

The type of the food matrix is of great importance for the thermal resistance of the tested microorganism (Soni et al. 2022). Therefore, data examined for one medium cannot be used for another. Food matrices may differ significantly, among others, in water content, pH value, amount of sugar, fat composition, and physical state—solid, liquid, semisolid (Soni et al. 2022). Consequently, scientists have developed numerous mathematical models, which are further discussed in Section 5.3, to incorporate these diverse factors into mathematical descriptions of microbial behavior.

**FIGURE 3:**
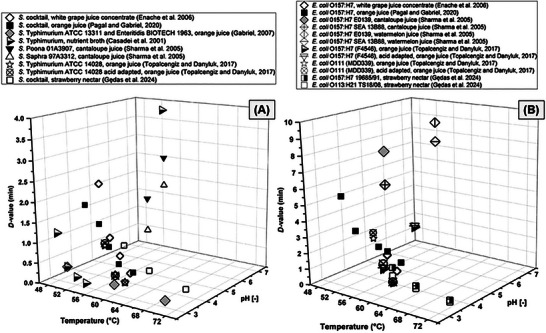
Summary of *D*‐values (min) for *Salmonella* spp. (A) and *E. coli* (B) strains in various juice and nectars.

In industry, the *D*‐ and *z*‐values of the selected target pathogens and the selected matrices are required to determine the parameters of thermal processes. For example, the International Fruit and Vegetable Juice Association recommends to use the data available in the Lemgo database (IFU 2024). Unfortunately, this database is not regularly updated and may not reflect the latest literature reports (https://ldzbase.de/index.php). For instance, the database contains no data on the thermal resistance of *Salmonella* spp. in fruit juices. The only data available is for skim milk, whole milk, beer, nonalcoholic beer, and CASO‐YE medium. Additionally, there is often no information about the *z*‐value. From the perspective of the fruit juice industry, the outdated nature of this database renders it questionable. The data summary in Figure 3 shows the *D*‐values of *Salmonella* spp. (Figure 3A) and *E. coli* (Figure 3B) strains in different fruit juices and nectars. The thermal resistance of microorganisms tested is in the temperature range of 48–72°C, however most of the reviewed literature concerns 56–60°C. Since the juices and nectars are characterized by low pH, most of the data on the thermal resistance of these pathogens are studied at pH 3.5–4.0. It is worth noting that in both cases, when there is less acidification of the environment, the bacteria show greater thermal resistance. For example, *S*. Poona 01A3907 and *E. coli* 0157:H7 E0139 in cantaloupe juice of pH 6.3 had *D*‐values at 57°C of 2.7 min and 8.2 min, respectively (Sharma et al. 2005). In any case, these data are crucial for optimizing the thermal preservation processes of juices and nectars, as they consider the variations in fruit composition and acidity levels.

Environmental factors may directly or indirectly affect the thermal resistance of bacteria. In the case of a direct impact, such as a low pH value of the matrix, cross‐protection may occur, which will improve the thermal resistance of the bacteria (Gabriel and Arellano 2014; Mazzotta 2001; Sharma et al. 2005). Interestingly, Gędas et al. (2024a) showed that the content of solid particles, rather than the pH value, has a greater influence on the obtained *D*‐values. For example, *E. coli* ATCC 8739 exhibited significantly higher *D*‐values in strawberry nectar containing fruit pulp than in the same nectar without it, while maintaining the same pH value and sugar content (Gędas et al. 2024a). Pathogens like *Salmonella* spp. can easily adapt to extreme conditions, which is why they can survive in low‐moisture products, such as chocolate, nuts, and peanut butter (Enache et al. 2017). Additionally, reduced water activity results in increased survival and thermal resistance of bacteria. For instance, Beuchat and Mann (2014) showed that a mixture of five *S. enterica* strains of different serotypes (Agona F5567, Enteritidis 2415, Montevideo G4639, Tennessee K4643, and Typhimurium DT104) were able to survive in dried strawberries for 6 months and in cranberries even up to 8 months when stored at 4°C. Thus, in low‐moisture foods, the moisture diffusion from the bacterial cells (Xie et al. 2021) plays a significant role in bacterial thermal resistance. Contrary, in high‐moisture foods, the composition of the matrix may affect heat transfer, thereby indirectly affecting microorganism inactivation. Yu et al. (2004) demonstrated that higher sugar concentrations resulted in a significant decrease in heat transfer due to the local increase of the solution saturation temperature. These changes in heat transfer are linked to other fluid properties, such as viscosity (Yu et al. 2004).

### 5.3 Mathematical Models for Calculating *D*‐Values

After experimentally testing the thermal resistance of microorganisms, the results must be described mathematically to obtain the necessary *D*‐ and *z*‐values. The first attempt to describe the nature of microbial inactivation was made by Bigelow (1921), who observed a logarithmic decline in the viable count over time. However, the classical determination of the thermal kinetics of a selected bacterium is only applicable to isothermal inactivation. For this reason, more complex models were developed that account for the relative effect of pH in the environment (Mafart and Leuguerinel 1998) as well as the combined effects of pH and water activity (Gaillard et al. 1998). A significant limitation of these models is the lack of consideration of the effect of variability (Georgalis et al. 2022). Microbial inactivation is often not strictly linear and typically occurs with deviations from first‐order kinetics. However, when the deviation is substantial, a nonlinear approach, such as the biphasic model (Cerf 1977), is recommended. It assumes that a population of microorganisms consists of two different subpopulations with different thermal resistance. For this reason, it can adapt to a lack of linearity in the inactivation curve (Akkermans et al. 2020). Moreover, the biphasic behavior can also be caused by the production of heat shock proteins, even in single strain populations (Humpheson et al. 1998). Another widely used nonlinear model is the Weibull model, which can predict thermal inactivation by modeling the statistical distribution of inactivation times (van Boekel 2002). Several scientists used this tool to select a surrogate for target pathogens (Fenoglio et al. 2020; Pokhrel et al. 2017). It can also be used for modeling microbial inactivation by nonthermal treatments, such as PEFs or high hydrostatic pressure (Buzrul 2022). However, this model is not perfect because it is not possible to add a tailing effect in this case (Akkermans et al. 2020). The model proposed by Geeraerd et al. (2000) considers tailing behavior and/or a shoulder effect. This situation may occur when dealing with a mixture of strains, such as in a bacterial cocktail, or it might indicate that a part of the population is more resistance to the applied treatment (Akkermans et al. 2020). For instance, the study of Georgalis et al. (2022) demonstrated differences in thermal resistance of two *Salmonella* spp. strains, where *S*. Senftenberg CECT 4565 showed a nonlinear inactivation and was more resistant than *S*. Enteritidis CECT 4300, which had a log‐linear response. However, Peleg (2000) examined the phenomenon of “shoulders,” referring to the initial flat regions observed before the decline in microbial survival curves, and proposed that such curves can be interpreted as the cumulative distribution of lethal events across a population with variable resistances. Computer‐simulated survival curves further demonstrated that the shape of the curve alone is insufficient to confirm a specific inactivation mechanism (Peleg 2000). Currently, a very large number of models and their variations are published, the main ones, which were just discussed, are presented in Table 3. Akkermans et al. (2020) provided a comprehensive overview of secondary models for thermal inactivation, tailored to specific individual microorganisms with defined environments, as the thermal resistance of a microorganism is significantly influenced by its surrounding matrix (see Section 5.2). These models consider external factors including pH, water activity, and the addition of antimicrobials such as carvacrol or cinnamaldehyde (Tchuenchieu et al. 2022; Gaillard et al. 1998; Juneja et al. 2001; Cerf et al. 1996). Incorporation of the impact of water content into the model is particularly critical for products like milk powder (Wei et al. 2020). Moreover, Corradini and Peleg (2009) proposed a dynamic heat inactivation model that includes bacterial heat adaptation into survival kinetics. Integrating an adaptation factor that changes over time as bacteria experience heat stress leads to more accurate predictions of microbial inactivation. This model includes stress adaptation, whereby bacteria can gradually increase their heat resistance during the process. Although the model was initially fitted using *E. coli* and *L. monocytogenes*, it may improve the accuracy of microbial risk assessments and process validation in thermal food preservation (Corradini and Peleg 2009). However, for thermal processes focused on ensuring food safety and stability, simpler models are typically preferred over complex mechanistic models (Smelt and Brul 2014).

**TABLE 3:** Overview of primary inactivation models (based on: Akkermans et al. 2020; Smelt and Brul 2014).

Model name	Equations	Description	References
Bigelow's model—exponential	$\log N\left(t\right)=\log N_{0\;}-\frac{t}{D_{T}}$	Heat inactivation—linear relation between logarithm of the number of microorganism (*N*) and the treatment time (*t*)	Bigelow 1921; Huertas et al. 2021
Extended Bigelow's model	$\log D=\log D^{*}\;-\;\frac{T\;-\;T^{*}}{z}-\;{\left(\frac{\mathrm{pH}\;-\;pH^{*}}{z_{\mathrm{pH}}}\right)}^{2}$	Bigelow model with the effect of pH	Mafart and Leguerinel 1998
Extended Mafart and Leguerinel's model	$\log D=\log D^{*}\;-\;\frac{T\;-\;T^{*}}{z}\;-\;{\left(\frac{\mathrm{pH}\;-\;pH^{*}}{z_{\mathrm{pH}}}\right)}^{2}\;-\;\frac{a_{w}-1}{z_{a_{w}}}$	Bigelow model with the effect of pH and water activity	Gaillard et al. 1998
Biphasic model	$\frac{dN_{1}\left(t\right)}{dt}=-\;k_{1}\;\times \;N_{1}\left(t\right)$ $\frac{dN_{2}\left(t\right)}{dt}=-\;k_{2}\;\times \;N_{2}\left(t\right)$	Assumption: microbial population has two subpopulations with a different inactivation rate; there are two equations for two subpopulations	Cerf 1977
Geeraerd model	$\frac{dN(t)}{dt}=-f_{\mathrm{shoulder}}\left(t\right)\times k_{\max}\left(t\right)\times f_{\mathrm{tail}}\left(t\right)\times N\left(t\right)$	Model for microbial inactivation with a shoulder and/or tailing effect; *k*_max_ (*t*) is the maximum specific inactivation; the *f*_shoulder_ (*t*) and *f*_tail_ *(t)* factors are calculated according to Akkermans et al. 2020	Geeraerd et al. 2000
Weibull model	$n\left(t\right)=\;n_{0}\;-\;{\left(\frac{t}{\delta }\right)}^{p}$	Based on the probability distribution of microbial death; the shape parameter (*p*) describes the cells nature (adapted to stress/damaged); δ corresponds to the *D*‐value	Peleg and Cole 1998; van Boekel 2002
Extended Weibull model	$\frac{dn(t)}{dt}=-\;\frac{p\;\times \;t^{p\;-\;1}}{\delta^{p}}\;\times \;\left(1\;-\;10^{n_{\mathrm{res}}\;-\;n\left(t\right)}\right)$	Based on the Weibull model including the tailing effect	Albert and Mafart 2005
Extended Arrhenius equation	$\ln\left(k\right)=\;a_{0}+\frac{a_{1}}{T}+a_{2}\;\times \;\mathrm{pH}+a_{3\;}\times \;pH^{2}$	Proposed for the effect of pH on *Clostridium botulinum* inactivation kinetics	Davey et al. 1978
Extended Davey's equation	$\ln\left(k\right)=\;a_{0}+\frac{a_{1}}{T}+a_{2}\;\times \;\mathrm{pH}+a_{3}\;\times \;pH^{2}+a_{4}\;\times \;a_{w}^{2}$	Based on Davey's equation including effect of water activity	Cerf et al. 1996
The Weibullian‐log logistic (WeLL) model	$\frac{d\mathrm{lo}g_{10}S\left(t\right)}{dt}=-\ln\left\{1+\exp\{k[T\left(t\right)-T_{c}]\}\right\}\;\times \;n\;\times \;[-\;\frac{\mathrm{lo}g_{10}S\left(t\right)}{\ln\{1+\exp\left\{k[T\left(t\right)\;-\;T_{c}\}\right\}}$	Dynamic heat inactivation model that integrates bacterial heat adaptation by including a logistic adaptation factor	Corradini and Peleg 2009
Polynomial models—example	$\log D=a_{0}+a_{1}\;\times \;T+a_{2}\;\times \;F$	Example of the polynomial model to assess the impact of temperature and adjusted fat (*F*) levels on the *D*‐value of *Salmonella* in poultry	Juneja et al. 2001
Sapru model	$\frac{dN_{1}}{dt}=-\left(K_{d1}+K_{a}\right)N_{1}$	Describes bacterial spore activation during sterilization	Sapru et al. 1992

It is worth noting that in addition to mathematical models, there are also tools that are intended to facilitate modeling. Bevilacqua et al. (2015) presented some examples of them. The first one is GInaFIT, an Excel add‐in component developed by Geeraerd et al. (2005), thanks to which eight different models can be created, including log‐linear curves with the addition of a shoulder/tailing effect, or biphasic models. This freeware add‐in was designed to bridge the gap between developers of predictive modeling approaches and users with daily business in the food industry who are not familiar with or do not have access to advanced, nonlinear regression analysis tools (Geeraerd et al. 2005). This makes this tool especially user‐friendly. Besides, Bevilacqua et al. (2015) also point to Meat and Livestock Australia (MLA), a tool to help manufacturers evaluate whether their in‐house meat fermentation conditions are sufficient to inactivate *E. coli*. In predictive microbiology, a valuable tool is ComBase as well (Bevilacqua et al. 2015). It has a 50,000‐records database including information on microorganism behavior as well as growth and inactivation models. Another example of a tool for predictive microbiology is IPMP Global Fit (Huang 2017), which is modified USDA IPMP (Huang 2014). IPMP is a family of software tools developed for data analysis, designed for inverse analysis to estimate kinetic model parameters, such as growth rate and *D*‐value. It combines advanced data analysis algorithms with an intuitive, user‐friendly interface that guides users step by step in selecting appropriate models and initial parameter estimates, which are essential for performing nonlinear regression (Huang and Juneja 2025). Peleg (2024) reviewed extensively various microbial inactivation kinetic models and survival curve shapes. The author emphasizes the temporal distribution of microbial inactivation and argues that all existing survival curve models do not fully capture all relevant information (Peleg 2024).

### 5.4 Applicability of Laboratory Data to Industrial Conditions

As Soni et al. (2022) highlighted, upscaling from laboratory to commercial scale poses a major challenge. The question arises as to whether the same behavior of microorganisms can be expected at both scales. Researchers emphasize a common issue in publications related to testing the thermal resistance of bacteria, which is the lack of information on the come‐up time (Soni et al. 2022). Beginning experiments upon reaching the target temperature allows for a comprehensive examination of temperature‐specific impacts. To achieve this, experimental methods are chosen that enable rapid sample heating, such as thin glass capillaries or the thermoresistometer Mastia. With these methods isothermal inactivation is possible. However, industrial conditions are more dynamic, typically involving three main stages: heating, processing, and cooling (Huertas et al. 2021). Therefore, it is essential to consider whether, from this perspective, it is better to account for the come‐up time in experiments and regard this moment as the start of inactivation. The other challenge with the comparison of the obtained *D*‐values of bacteria is the difference in sample volume between laboratory settings and food processing conditions. Laboratory samples are typically much smaller and offer cost advantages due to reduced medium usage. The range of laboratory volumes of tested samples is also large, from 20 µL (Usaga et al. 2014a, 2014b) to 400 mL (Alvarez‐Ordonez et al. 2009b; Conesa et al. 2009; Huertas et al. 2021; Clemente‐Carazo et al. 2020). The impact of sample volume on thermal resistance data requires thorough investigation and consideration when translating laboratory findings to real‐world processing environments.

Smelt and Brul (2014) underscored the deficiency in publications on bacterial thermal resistance where only *D*‐values are reported without accompanying inactivation curves, thus limiting the possibility of microbial behavior analysis. Furthermore, not all researchers provide *z*‐values either, they only limit themselves to *D*‐values. Table 4 presents a comparison of exemplary *z*‐values of *Salmonella* spp. with recalculated values based on Formula 2. Interestingly, not all calculated *z*‐values correspond to the data given in the reference. This is heavily influenced by the values used for the calculation. For instance, Enache et al. (2006) reported that the *z*‐value of *Salmonella* cocktail in white grape juice concentrate is 8.8°C. However, the *z*‐value can be calculated from the *D*‐values given in the publication using Formula 2. The thermal resistance of the selected bacteria was indicated in this example for four temperatures: *D*_56_ = 2.51 min, *D*_58_ = 1.26 min, *D*_60_ = 0.87 min, and *D*_62_ = 0.50 min. Thus, if the entire range of tested temperatures will be considered, it maintains:

$$z=\frac{62^{\circ }C-56^{\circ }C}{\log2.51-\log0.50}=8.56^{\circ }C$$

**TABLE 4:** Overview and comparison of *z*‐values of *Salmonella* spp. from the literature and recalculated based on Formula 2.

Strains	Method	Range of tested temperatures (°C)	Medium	*z*‐value from the ref. (°C)	Calculated *z*‐value (°C)	References
*Salmonella* cocktail (*S*. Enteritidis ATCC 13076, *S*. Montevideo ATCC 8387, *S*. Newport ATCC 6962, *S*. Typhimurium ATCC 14028)	Polyethylene Whirl‐Pak sample bags (7.5 × 12.5 cm) immersed in a water bath	55–65	TSBYE	6.3	6.5	Kim et al. 2019
*S*. Typhimurium CECT 443	350 mL of juice heated in thermoresistometer TR‐SC	50–58	Orange juice	5.8	5.8	Alvarez‐Ordonez et al. 2009b
			Apple juice	5.7	5.7	
*S*. Senftenberg CECT 4384		55–63	Orange juice	6.9	6.8	
			Apple juice	7.2	7.3	
*S*. Typhimurium ATCC 14028	Filtered sample bags in a water bath—Linear model	75–95	Mash boiler feed	28.6	25.2	Coe et al. 2022
	Filtered sample bags in a water bath—Weibull model			21.8	28.0	
*Salmonella* cocktail (*S*. Rubislaw N‐4019, *S*. Gaminara N‐4020, *S*. Hartford N‐4021, *S*. Thomson N‐4023, *Salmonella* human isolate N‐4078)	250 mL sterile Erlenmeyer flask immersed in a water bath	56–62	White grape juice concentrate, 58 Brix, pH 3.3	8.8	8.6 (range 56–62°C) 8.7 (range 56–60°C) 10.0 (range 58–62°C)	Enache et al. 2006
*Salmonella* cocktail (*S*. Typhimurium ATCC 14028, *S*. Abortus‐Equi ATCC 9842, *S*. Enteritidis LFMH 10, *S*. Montevideo LFMH S3‐10, *S*. Diarizonae ATCC 12325 and 29934, *S*. Oranienburg S8‐18, *S*. Senftenberg S7‐18)	Glass tubes—9.9 mL of solution—immersed in a water bath	50–60	Orange juice Rauch Happy Day	12.35	13.5	Pagal and Gabriel 2020
*Salmonella* cocktail (*S*. Typhimurium NRRL B‐4420, *S*. Typhi NRRL B‐573, *S*. Enteritidis Biotech 1963)	Screw‐capped test tubes containing 5 mL test fruit beverages immersed in a water bath	60–73	Apple juice	No data	49.0	Gabriel et al. 2015
			Orange juice		42.7	
			Mango nectar		41.3	
			Soursop nectar		157.0	
			Guava nectar		35.3	
*Salmonella* cocktail (serovars Senftenberg, Typhimurium, Heidelberg, Mission, Montevideo, and California)	Inoculated meat samples (8.5 g) in thin‐wall (0.7 mm) metal containers immersed in a circulation water bath	55–70	Chicken patties	No data	7.8	McCormick et al. 2003
			Chicken tenders		8.1	
			Franks		9.5	
			Beef patties		9.6	
			Beef‐turkey patties		8.6	
*S*. Enteritidis ATCC 13076	Thermal‐death‐time disks	54–60	Liquid whole egg	4.08	3.0 (range 54–58°C) 3.9 (range 54–60°C) 5.9 (range 56–60°C) 10.0 (range 58–60°C)	Jin et al. 2008
		52–58	Liquid white egg	4.03	3.4 (range 52–56°C) 4.0 (range 52–58°C) 4.4 (range 54–58°C) 5.8 (range 56–58°C)	
*Salmonella* cocktail (*S*. Senftenberg LTH 5703, *S*. Typhimurium ATCC 13311 and ATCC 14028, *S*. Saintpaul LTH 6494, *S*. Enteritidis ATCC 13076)	Glass tubes—1.5 mL of solution—immersed in a water bath	60–72	Strawberry nectar	25.30	19.6 (range 60–65°C) 25.3 (range 60–72°C) 31.6 (range 65–72°C)	Gędas et al. 2024b

However, if a different temperature range from that is chosen, a different result can be obtained, namely:


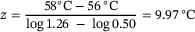




It is extremely important that the selected range of temperatures significantly changes the *z*‐value. In the available literature on this topic there is no clear recommendation regarding how large the temperature range considered for *z*‐value calculations should be. It is worth noting that there is also no single, leading, and recommended method for determining and calculating the parameters of thermal kinetics of microorganisms. This underscores the need for more fundamental research on heat resistance and transfer of these results into industrial settings. This would enable sound decisions on the intensity of treatments while taking into account consumer health, food safety, product quality, and process sustainability.

## 6 Conclusions

Thermal processing is fundamental to food processing and preservation. Its aim is to ensure the microbiological safety of food. However, high temperatures can reduce the nutritional quality of products, making optimization essential. Therefore, it is crucial to study the thermal resistance of relevant pathogenic bacteria. Due to the frequency of infections, the risk of hospitalization, and the outbreaks associated with fruits and fruit products, the emphasis was on *Salmonella* spp. and *E. coli* as target pathogens. Contamination routes were highlighted, emphasizing the critical points in the production of fruit products.

In this review, the process of selecting surrogate microorganisms was presented with a focus on the most problematic aspects. Currently, there are no standardized guidelines for research methodology, leading to significant discrepancies. This can hinder efforts to optimize pasteurization processes in the food industry, potentially compromising product safety and shelf life. Standardizing methods would facilitate to achieve consistency in *D*‐value determination, ultimately leading to more reliable risk assessments and better process design. The selection of an appropriate testing method largely depends on the type of product being tested. For example, glass tubes and capillaries are suitable for liquid foods, while sealed pouches or Stomacher bags are used for meat products. By presenting available research methods, discussing their advantages and disadvantages, and indicating their frequency of use by researchers, we recommend the use of glass capillaries for liquid foods such as fruit juices. The influence of the matrix on the bacterial thermal resistance is huge. Understanding how different matrices affect the thermal resistance of specific bacterial species will contribute toward optimizing thermal processes to ensure both safety and quality in food products. That is why experimental research adapted to the requirements of the process is so important. Additionally, this review provides a comprehensive overview of calculating *D*‐ and *z*‐values. A research gap is demonstrated regarding the lack of a clear indication of the temperature range for calculating *z*‐values and the consequent problems resulting from it. To address this gap, we recommend that future research focuses on standardizing and identifying the optimal temperature range that most accurately reflects bacterial behavior, thereby improving the reliability of thermal resistance data. There are a large number of models describing the thermal inactivation of microorganisms mathematically. These models provide additional information on specific survival mechanisms. However, they also create challenges in applying this data to the food processing environment, which highlights the need for further research to better integrate scientific findings with practical applications in food safety. In the long term, this will enable better integration of existing research findings, more targeted identification of new research needs in the field of bacterial thermal resistance, and, from an application perspective, the optimization of food preservation processes, ultimately resulting in safer and less‐processed food products.

## Author Contributions

**Astrid Gędas**: conceptualization, writing – original draft. **Agnes Weiss**: writing – review and editing, funding acquisition.

## Funding

This project has received funding from European Union's Horizon 2020 research and innovation program under the Marie Skłodowska‐Curie grant agreement No. 956257.

## Conflicts of Interest

The authors declare no conflicts of interest.
